# International trade and unemployment: towards an investigation of the Swiss case

**DOI:** 10.1186/s41937-017-0006-7

**Published:** 2018-06-05

**Authors:** Lukas Mohler, Rolf Weder, Simone Wyss

**Affiliations:** Faculty of Business and Economics, University of Basel, Peter Merian-Weg 6, 4002 Basel, Switzerland

**Keywords:** International trade, Unemployment, Low-skilled labour, Switzerland, F14, F16

## Abstract

The topic of this paper has been motivated by the rising unemployment rate of low-skilled relative to high-skilled labour in Switzerland. Between 1991 and 2014, Switzerland experienced the highest relative increase in the low-skilled unemployment rate among all OECD countries. A natural culprit for this development is “globalization” as indicated by some mass layoffs in Switzerland and as commonly voiced in public debates all over the world. Our analysis, which is based on panel data covering the years 1991 to 2008 and approximately 33,000 individuals employed in the Swiss manufacturing sector, does not, however, confirm this presumption. We do not find strong evidence for a positive relationship between import competition and (low-skilled) individuals’ likelihood of becoming unemployed.

**Electronic supplementary material:**

The online version of this article (10.1186/s41937-017-0006-7) contains supplementary material, which is available to authorized users.

## Introduction

The relationship between international trade and employment has always been controversial. Trade economists have traditionally emphasized the efficiency-enhancing effects of international trade with no impact on total employment, at least in the medium and long term. Politicians and members of governments, in contrast, typically believe in an employment-increasing effect of international trade and often point to the numbers of jobs created by rising exports.^1^ In the eyes of the public, however, international trade entails the danger of job destruction, particularly through increased imports. Trade economists agree that international trade may have distributional effects within countries. But they typically identify these effects in terms of changing factor prices: Low-skilled labour may, for example, lose ground—relatively and absolutely—in a high-income country as a result of international trade with (low-skilled) labour-abundant countries such as China or India.

In this paper, we investigate whether international trade is indeed linked to the likelihood of becoming unemployed. The focus on unemployment is motivated by our observation that the Swiss unemployment rate between low-skilled labour and high-skilled labour increased faster than that of any other OECD country between 1991 and 2014, with virtually no change in the relative wage rate between the same two groups of people. We use a representative panel data set for employees in the Swiss manufacturing sector, covering the period from 1991 to 2008, and link it to international trade data. We control for a number of individual characteristics, particularly regarding skills, age and experience, as well as industry properties. The analysis indicates that, for the Swiss economy, rising or high levels of imports do not seem to be a driving force behind the probability of becoming unemployed. Individual characteristics such as a short length of tenure, part-time employment, and low skills are, however, confirmed to be important factors that positively affect the individual’s risk of becoming unemployed.

Thus, the paper adds to the rapidly expanding literature on whether international trade is an important cause of the increase in the wage and unemployment gaps between skilled and unskilled labour that have been observed in the USA and some other countries since the 1980s.^2^ We know since Stolper and Samuelson (1941) and, more generally, since Jones (1965) that trade liberalization tends to have a strong negative impact on some real factor prices and, if these are inflexible or search costs are involved, also on factor market clearing, as shown by Davis (1998b), Davidson et al. (1999), and Egger and Kreickemeier (2008). Moreover, Feenstra and Hanson (2003) argue that the effects from trade in intermediate inputs may be similar to those caused by skill-biased technological change which is often made responsible for the wage gap in the US economy. Autor et al. (2013) found significant negative labour-market effects on the US economy of international trade between the USA and China and conclude: “Rising imports cause higher unemployment, lower labor force participation, and reduced wages in local labor markets that house import-competing manufacturing industries” (p. 2121).

Recent trade models, which introduce some labour market frictions, as used by Brecher and Chen (2010), Davis and Harrigan (2011), Helpman and Itskhoki (2010), Helpman et al. (2010), Larch and Lechthaler (2011), Mitra and Ranjan (2010), or Ranjan (2012), imply that relative unemployment between different types of labour may be affected by trade liberalization in a variety of ways. Moreover, these models come to the conclusion that international trade may also affect the overall unemployment level in an economy—positively or negatively.^3^ In empirical analyses, a negative effect of trade on overall unemployment is found by Felbermayr et al. (2011) and by Gozgor (2014) in cross-country analyses, by Hasan et al. (2012) for India and by Francis and Zheng (2011) for NAFTA.^4^ Chusseau et al. (2010)—in a cross-country analysis—and Horgos (2012)—for Germany—show that in the case of inflexible factor prices an increase in the relative unemployment rate between skilled and unskilled labour can to some extent be linked to trade—which the former call an “inequality-unemployment trade-off”. Fugazza et al. (2014) find a positive relationship between trade and unemployment in a panel of 97 countries if countries have “a comparative advantage in sectors that have high labour market frictions” (p. 1).

Compared to the existing literature, our empirical investigation is of particular interest for three reasons. First, it focuses on a small country whose international trade reflects a large share of its domestic output. The Krugman (2000) critique that a country’s trade volume is typically too small to explain effects on different types of labour hardly applies in this case (or at least to a much lesser extent). Second, our paper’s emphasis is on the unemployment rate, and not on wages as underlined by the majority of empirical research studies.^5^ This focus is in line with the recent shift in research interest among trade theorists and labour-market economists as well as with the stylized facts applying to the Swiss economy. Finally, we add to the limited literature on Switzerland in this field. The relationship between international trade and unemployment has, to our knowledge, not been analysed to date for the Swiss case.^6^

The remainder of the paper is as follows. The “Background” section presents stylized facts that explain our research strategy. The “Methods” section briefly describes our research methodology. The “Results and discussion” section presents the main results of the econometric analysis. The “Conclusions” section concludes.

## Background

Past research has been motivated by an inquiry into the impact of international trade on *relative wages*. Feenstra (2010, pp. 10), for example, describes and discusses the development of the wages of “nonproduction” relative to “production” workers in US manufacturing from 1958 to 2006. If we interpret this ratio as the relative wage rate of high-skilled to low-skilled labour, the data clearly shows that the relative wages of unskilled labour fell considerably and constantly from 1986 to 2000. This observation has been the basis for the expanding literature on trade and the wage gap in the USA that also sparked our research interest with its focus on Switzerland.

**Fig. 1:**
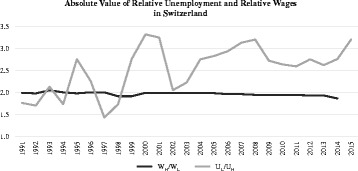
Evolution of relative wages and relative unemployment in Switzerland. Source: Own calculations based on FOS (2008), Wyss (2010) FOS (2016a, b)

Such a development is, however, not observable for Switzerland. Using Swiss labour market panel data (Swiss Labor Force Statistic, SLFS) and the UNESCO skill classification scheme (International Standard Classification of Education, ISCED-97),^7^ we calculated both the median gross wage rate of high-skilled (*W*_H_) and low-skilled (*W*_L_) labour, and the unemployment rate for the same two groups, i.e. *U*_H_ and *U*_L_, for the period 1991 to 2014. Figure 1 shows that, over this period, the *U*_L_/*U*_H_ rose with a compounded annual growth rate (CAGR) of 2%, whilst the *W*_H_/*W*_L_ remained roughly constant with a CAGR of − 0.3%. Thus, Fig. 1 serves as a motivation to study a possible relationship between international trade and (changes in) the relative unemployment of low-skilled and high-skilled labour in the Swiss case.

**Fig. 2:**
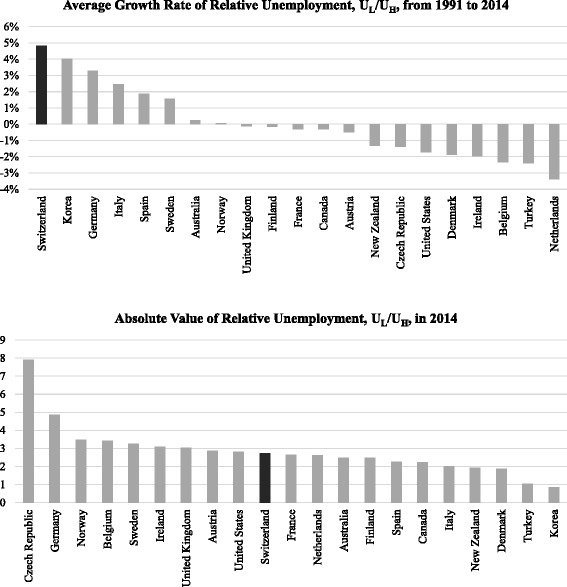
Average growth rate of relative unemployment (top panel, 1991–2014) and absolute value of relative unemployment (bottom panel, 2014) in OECD countries. Note: These are OECD countries for which data were available for the years considered. For the comparison in the top-panel, compounded average growth rates were taken. Source: Own calculations based on OECD (2007) and OECD (2015), Tables A8.4a and A5.4a, respectively

A comparison among 21 OECD countries implies that there is no other country in which *U*_L_/*U*_H_ has grown as fast as in Switzerland from 1991 to 2014.^8^ Figure 2 shows a CAGR of 4.8% of this ratio from 1991 to 2014 (top panel). It reveals that other countries such as South Korea or Germany also experienced a large rise in this ratio, whereas countries like the Netherlands or Belgium but also the USA or Canada demonstrate a decrease of the relative unemployment of low-skilled labour. Absolute numbers in the OECD data indicate that the Swiss *U*_L_ increased from 1.2% (1991) to 8.8% (2014), whereas *U*_H_ increased to a much smaller extent over this period (from 1.3 to 3.2%). Note, however, that the absolute value of the relative unemployment rate in Switzerland (2.7) is not extremely high, but rather puts the country in the middle of the reported OECD countries as shown in Fig. 2 (bottom panel). Given the strong and yet unbroken trend in the Swiss relative unemployment rate, it is of highest interest to assess whether trade may be a driving force of this development.^9^

## Methods

Trade theory stresses the importance of international trade in improving an economy’s *allocation of resources,* and *not* the creation of additional jobs. In a standard trade model, there is no expected link between trade liberalization and the total number of jobs in an economy.^10^ The argument trade economists traditionally have put forward is that whilst more trade leads to some jobs being destroyed in the import-competing sector of an economy, new jobs are simultaneously being generated in the export sector.

An increase in unemployment is, however, compatible with the traditional trade theory if we, for example, extend a Heckscher-Ohlin type model to allow for some factor price inflexibility as shown by Davis (1998b) or, adding trade in intermediate inputs, by Egger and Kreickemeier (2008). The reason is that trade typically leads to a decrease in the relative demand for low-skilled labour in a (human) capital-rich country. If the induced fall of the price of low-skilled labour—predicted by the Stolper Samuelson Theorem—is prevented by labour market rigidities, unemployment for low-skilled labour tends to rise with trade liberalization.

Recent trade models expanded in this direction allowing for a number of labour market frictions and/or using intra-industry trade models based on heterogeneous firms and job-specific rents. It turns out that, in these set-ups, trade liberalization may indeed raise unemployment of particular types of labour and affect overall unemployment in an economy. In Brecher and Chen (2010), for example, the unemployment rates of low- and high-skilled labour “often move in opposite directions” (p. 990), whereas the change of aggregate unemployment is ambiguous. Davis and Harrigan (2011) argue that, in their model, trade liberalization may destroy a considerable share of highly paid jobs without, however, necessarily affecting overall unemployment. Helpman and Itskhoki (2010, p. 1100) find the surprising result that “[T]he opening to trade raises a country’s rate of unemployment if its relative labour market frictions in the differentiated sector are low.” And Hasan et al. (2012, p. 269) come, based on their empirical study of India, to the conclusion: “Moreover, our industry-level analysis indicates that workers in industries experiencing greater reductions in trade protection were less likely to become unemployed, especially in net exporting industries.”^11^

The focus of our paper is empirical. We seek to explain the employment status of individuals over time, i.e. whether they become unemployed or not, by changes and levels of imports and exports, controlling for various individual characteristics and industry factors. The explained variable (i.e. the individual’s status, *y*_*i*_) is qualitative in nature and takes a value of 1 if an individual becomes unemployed in a certain year and 0 otherwise. The explanatory variables will be qualitative or quantitative as will be made more precise in the “Results and discussion” section. The econometric analysis of the relationship between the two is largely based on the linear probability model (OLS) that includes year and industry fixed effects and, for some specifications, individual fixed effects. We use this model as coefficients will be easier to interpret, but we also report the results of the analysis based on the logit model. They turn out to be qualitatively the same.

## Results and discussion

We base our analysis on representative industry-panel data for the years 1991 to 2008. During this period, Switzerland established a number of bilateral agreements with trading partners—including the European Union (EU). Moreover, mutual trade liberalization between Switzerland and other countries also occurred through new membership of countries to the World Trade Organization (WTO), the EU and the European Free Trade Association (EFTA).^12^ All of this implies pressure and adjustments that are typical for trade liberalizations. The question we now seek to answer is whether international trade indeed had a significant impact on the probability of (particularly low-skilled) individuals to become unemployed. If this is the case, international trade could be one reason for the increase of the relative unemployment rate for low-skilled labour described in the “Background” section.

Using micro data on individuals’ characteristics, we intend to assess whether an individual, who becomes unemployed, does so because of his or her particular exposure to international trade, controlling—amongst others—for skills. We present detailed summary statistics of the underlying data in the “The data” section and then run regressions of the change in the individual employment status on individuals’ characteristics and the trade variables in the “Changes in employment status, individual characteristics and trade” section. The “Refinement of the trade variables and inclusion of individual fixed effects” section uses a number of refined trade variables and includes individual fixed effects. The “Sensitivity analyses” section concludes with some sensitivity analyses.

### The data

For the industry panel data, we rely on the Swiss Labour Force Survey (SLFS). It is based on an annual and representative collection of information from Swiss residents (including foreigners, but excluding cross-border commuters) by the Swiss Federal Office of Statistics (FOS). The SLFS is in line with the methods used by the International Labour Office (ILO) which defines those individuals as unemployed who are not working, but searching for a job and ready to assume employment quickly.

This data source includes a pool of roughly 33,000 individuals over a period of 18 years (1991–2008) who were employed in the secondary sector (manufacturing) in Switzerland. As we want to attribute an industry to an individual, characterizing in which kind of industry the worker is employed, we link the SLFS data (FOS, 2009a) on the industry two-digit SIC level with the Swiss Foreign Trade Statistics (EZV, 2009) and the National Account Statistics of the FOS (2009b). To also characterize whether an individual works in a so-called ICT industry (i.e. an industry which displays an above-average intensity in the use of information and communication technology) or in a GAV industry (i.e. an industry which shows an above-average coverage of collectively bargained labour contracts), we also take into account the ICT-Survey of the KOF Swiss Economic Institute (KOF, 2005) at the Swiss Federal Institute of Technology (ETH) and the GAV-Statistics of the FOS (2002).

Summary statistics of the data used in our regressions are provided in Table 1. The first column entitled “Change in Employment Status” is composed of individuals who are either employed during the full period of observation or indicate a change in their employment status from employment to unemployment. The second column “Employment Status” includes all individuals with a status of employed or unemployed. This leads to a maximum of 20,928 (40,875) observations of which 463 (1226) show a change in the employment status from employed to unemployed (show a status of unemployment). These observations stem from 10,242 (18,995) individuals, of which 461 (1008) show a change in their status from employed to unemployed (show at least once a status of unemployment).^13^

**Table 1:** Summary statistics of the regression data set

Dependent variable	Change in employment status	Employment status
	(1)	(2)
Observations and individuals:		
No. of observations	20,928	40,875
With status “becoming unemployed”	463	
With status “being unemployed”		1226
No. of observed individuals	10,242	18,995
Of which becoming unemployed at least once	461 (733 obs.)	
Of which being unemployed at least once		1008 (2838 obs.)
Mean no. of observations/individual	2.0	2.2
Trade covariates:		
Mean annual import changes	6.9%	6.3%
Median annual import changes	5.8%	4.6%
Mean annual export changes	7.6%	7.1%
Median annual export changes	6.9%	6.4%
Industry characteristics:		
ICT intensive	37.2%	36.3%
Not ICT intensive	62.8%	63.7%
GAV sector	40.1%	39.6%
Non-GAV sector	59.9%	60.4%
Worker and job characteristics:		
Mean age	42.6	41.2
High-skilled	25.2%	23.9%
Medium-skilled	52.1%	52.5%
Low-skilled	22.8%	23.5%
Swiss citizen	60.6%	59.0%
Foreigner	39.4%	41.0%
Male	70.4%	69.0%
Female	29.6%	31.0%
Single	24.4%	27.5%
Married	64.0%	61.2%
Widowed	1.7%	1.6%
Divorced	10.0%	9.7%
Full-time	86.4%	85.7%
Part-time	13.6%	14.3%
Fixed contract	98.6%	97.2%
Temporary	1.4%	2.8%
Short tenure (< 1 year)	2.5%	11.3%
Medium tenure (1 to < 5 years)	29.3%	29.0%
Long tenure (> 5 years)	68.3%	59.7%

Source: Panel data set constructed using data from FOS (2009a), EZV (2009), KOF (2005) and FOS (2009b). Note that trade covariates and industry characteristics describe the industry which an individual is employed in

Our main econometric analyses will concentrate on the observations reported in the first column of Table 1. However, we will take into account the observations in the second column in our sensitivity analysis (“Sensitivity analyses”). Regarding the first column, the mean year-to-year change in percentage of import (export) values in the 17 manufacturing industries considered in the analysis amounts to 6.9% (7.6%). 40.1% of the observations are linked with “GAV industries”, whereas 37.2% of the observations include individuals employed in “ICT industries”.^14^ The distribution of the observed worker characteristics are reported in the bottom part of Table 1 and speak for themselves.

### Changes in employment status, individual characteristics and trade

We first regress changes in the individual employment status on the individuals’ characteristics and aggregate trade variables, using the following linear probability model with time and industry fixed effects:

1 $$ {y}_{it}=\alpha +{\beta}_1{ICT}_i+{\beta}_2{GAV}_i+{\beta}_3{SDF}_{it}+{\beta}_4{IM}_{it}+{\beta}_5{EX}_{it}+{\varepsilon}_{it}. $$

Note that *i* indexes the individual and *t* the year. The left-hand variable, *y*_*it*_, takes the value of 1 if the individual *i* becomes unemployed in *t* and was employed in *t − 1,* and it takes the value of 0 if the individual remains employed in *t*. The probability of becoming unemployed over time is explained based on a number of right-hand independent variables, starting with an individual being employed in an ICT and GAV industry, a number of socio-demographic factors (SDF) of individual *i* in *t* as well as imports (*IM*) and exports (*EX*) of the industry, in which the individual *i* is employed, in time *t*. Note that we use levels (i.e. the value) as well as changes (i.e. in percentage) for the trade covariates and also include lags. We also interact some of the variables with the individuals’ skill level (L, M, H). The results are provided in Table 2.

**Table 2:** Linear regressions of changes in employment status on trade variables and individual characteristics

Dependent variable: change in employment status					
	No trade covariates	Trade levels	Trade levels, lagged	Trade first diff.	Trade first diff., lagged
	(1)	(2)	(3)	(4)	(5)
Trade covariates					
Imports		0.012	0.010	0.006	0.025
		(0.019)	(0.019)	(0.028)	(0.027)
Exports		− 0.001	− 0.002	0.013	− 0.014
		(0.019)	(0.019)	(0.030)	(0.025)
Imports*low-skilled		0.017**	0.016**	− 0.002	− 0.002
		(0.008)	(0.007)	(0.060)	(0.042)
Imports*medium-skilled		0.002	0.001	0.007	− 0.027
		(0.005)	(0.005)	(0.036)	(0.024)
Exports*low-skilled		− 0.011*	− 0.011*	− 0.004	− 0.009
		(0.006)	(0.006)	(0.064)	(0.028)
Exports*medium-skilled		− 0.002	− 0.001	− 0.009	0.012
		(0.004)	(0.004)	(0.029)	(0.024)
Industry characteristics					
ICT intensive	− 0.005	0.001	0.002	− 0.006	− 0.009
	(0.023)	(0.026)	(0.027)	(0.023)	(0.012)
ICT intensive*low-skilled	− 0.001	0.000	0.000	− 0.001	− 0.001
	(0.008)	(0.008)	(0.008)	(0.008)	(0.008)
ICT intensive*medium-skilled	− 0.000	0.000	0.000	− 0.000	0.000
	(0.005)	(0.005)	(0.005)	(0.004)	(0.005)
GAV	− 0.015	− 0.021	− 0.013	− 0.016	− 0.011
	(0.019)	(0.044)	(0.044)	(0.019)	(0.017)
GAV*low-skilled	0.009	0.012*	0.012*	0.009	0.009
	(0.006)	(0.007)	(0.007)	(0.006)	(0.006)
GAV*medium-skilled	0.002	0.002	0.002	0.001	0.001
	(0.005)	(0.005)	(0.005)	(0.005)	(0.005)
Worker and job characteristics					
Low-skilled	0.013**	− 0.000	0.001	0.013*	0.014**
	(0.006)	(0.006)	(0.006)	(0.008)	(0.007)
Medium-skilled	0.006**	0.006	0.007	0.006*	0.007**
	(0.003)	(0.006)	(0.006)	(0.004)	(0.003)
Foreigner	0.010**	0.010**	0.010**	0.010**	0.010**
	(0.004)	(0.004)	(0.004)	(0.004)	(0.004)
Age	− 0.003**	− 0.003**	− 0.003**	− 0.004**	− 0.004**
	(0.001)	(0.001)	(0.001)	(0.001)	(0.001)
Age^2	0.000***	0.000***	0.000***	0.000***	0.000***
	(0.000)	(0.000)	(0.000)	(0.000)	(0.000)
Female	0.001	0.001	0.001	0.001	0.001
	(0.003)	(0.003)	(0.003)	(0.003)	(0.004)
Married	− 0.009***	− 0.009***	− 0.009***	− 0.008***	− 0.008***
	(0.003)	(0.003)	(0.003)	(0.003)	(0.003)
Widowed	− 0.019**	− 0.019**	− 0.019**	− 0.018**	− 0.018**
	(0.008)	(0.008)	(0.008)	(0.008)	(0.007)
Separated	0.007**	0.007**	0.008**	0.008**	0.008**
	(0.004)	(0.004)	(0.004)	(0.004)	(0.004)
Part-time worker	0.012**	0.011**	0.011**	0.012**	0.012**
	(0.005)	(0.005)	(0.005)	(0.005)	(0.005)
Temporary worker	0.113***	0.113***	0.112***	0.113***	0.113***
	(0.030)	(0.030)	(0.030)	(0.030)	(0.030)
Short tenure (< 1 year)	0.202***	0.202***	0.206***	0.205***	0.205***
	(0.048)	(0.048)	(0.049)	(0.049)	(0.049)
Medium tenure (1 to < 5 years)	0.017***	0.017***	0.017***	0.017***	0.017***
	(0.006)	(0.006)	(0.006)	(0.006)	(0.006)
Constant	0.478*	0.457*	0.496*	0.080**	0.078**
	(0.267)	(0.270)	(0.283)	(0.039)	(0.039)
Number of observations	20,928	20,928	20,895	20,878	20,866
Adjusted *R*^2^	0.086	0.086	0.088	0.089	0.087

Note: All regressions including year and industry fixed effects

Source: Panel data set constructed using data from FOS (2009a), EZV (2009), KOF (2005) and FOS (2009b)

*p < 0.10, **p < 0.05, ***p < 0.01

We start with a base regression, leaving out all trade variables. The results are reported in the first column of Table 2. They show that the likelihood of becoming unemployed significantly depends on the individual’s qualifications (medium and low skills) and type of contract (part-time, temporary contract).^15^ In this respect, we find also a positive relationship between the individuals’ likelihood of becoming unemployed and a short or medium tenure and for foreigners (typically due to a lack of local language skills). Married and widowed employees, on the other hand, are associated with a lower probability of becoming unemployed. Note that the coefficient for employment in an ICT-intensive industry or in a GAV industry is not significantly different from zero. The size of the coefficients in Table 2 can be interpreted as follows: Compared to a high-skilled worker, a low-skilled employee bears a 1.3% higher probability of becoming unemployed.

Columns (2) to (5) include levels and changes in the trade variables (*IM*, *EX*), also interacted with individuals’ skill levels (low-skilled, medium-skilled). Trade levels enter the estimation in logs, whereas “trade first differences” are calculated as the rate of year-to-year changes in percentage. We also add lagged trade variables (lagged by 1 year) to allow for a more deferred adjustment process. Note that, overall, the coefficients of worker and job characteristics do not change in a qualitative manner in these different specifications, nor do the GAV and ICT coefficients (except for the low skill level as a consequence of its interaction with the trade variables). We find some evidence (on the 5% significance level) for a significant effect of import *levels* on the probability of becoming unemployed for low-skilled employees: A 1% higher import value is associated with a 0.017% (0.016% for lagged imports) higher probability of becoming unemployed. In other words, low-skilled individuals who work in industries characterized by relatively large contemporaneous imports may, ceteris paribus, face a slightly greater likelihood of becoming unemployed. As shown in the fourth and fifth columns of Table 2, no significant effects are found for first differences (i.e. *changes*) in import and export values: A change in imports or exports in a certain industry does not significantly affect the probability of becoming unemployed.

We further investigate the impact of trade in the next subsection by using more refined trade variables and by including individual fixed effects to take into account any unobserved individual characteristics.

### Refinement of the trade variables and inclusion of individual fixed effects

We now regress changes in the individual employment status on a number of trade variables, distinguishing between imports in finished and intermediate products and between trade with the North and the South.^16^ We eliminate individuals’ characteristics as well as the GAV and ICT variables as we now use individual fixed effects.^17^ We continue applying the linear probability model with time fixed effects. Standard errors are clustered by industry. We start with taking trade levels (in logs) as explanatory variables and then proceed to look at the rates of changes of the same variables. The results are reported in Tables 3 and 4.

**Table 3:** Linear regressions of changes in employment status on trade levels using individual fixed effects

Dependent variable: change in employment status				
	Trade levels		Trade levels, lagged	
	(1)	(2)	(3)	(4)
Imports, total	0.016		− 0.002	
	(0.022)		(0.019)	
Exports, total	− 0.036*		0.004	
	(0.018)		(0.013)	
Imports, final prod., north		0.013		0.003
		(0.014)		(0.015)
Imports, interm. prod., north		− 0.003		0.006*
		(0.005)		(0.003)
Imports, final prod., south		0.002		0.008***
		(0.003)		(0.003)
Imports, interm. prod., south		0.002		− 0.000
		(0.006)		(0.003)
Exports, final prod., north		− 0.002		0.001
		(0.012)		(0.013)
Exports, interm. prod., north		− 0.019*		− 0.012
		(0.009)		(0.009)
Exports, final prod., south		− 0.009		0.001
		(0.008)		(0.006)
Exports, interm. prod., south		0.010**		− 0.001
		(0.004)		(0.004)
Constant	0.149***	0.118***	0.100**	0.102**
	(0.044)	(0.028)	(0.043)	(0.045)
Number of observations	20,928	19,438	20,895	19,406
Adjusted *R*^2^	0.045	0.047	0.045	0.047

Note: All regressions including time and individual fixed effects

Source: Panel data set constructed using data from FOS (2009a), EZV (2009), KOF (2005) and FOS (2009b)

*p < 0.10, **p < 0.05, ***p < 0.01

**Table 4:** Linear regressions of changes in employment status on trade differences using individual fixed effects

Dependent variable: change in employment status				
	Trade first differences		Trade first differences, lagged	
	(1)	(2)	(3)	(4)
Imports, total	0.013		− 0.009	
	(0.018)		(0.015)	
Exports, total	− 0.025		0.010	
	(0.015)		(0.007)	
Imports, final prod., north		0.008		0.002
		(0.014)		(0.015)
Imports, interm. prod., north		− 0.004		− 0.005*
		(0.004)		(0.003)
Imports, final prod., south		− 0.000		0.004**
		(0.001)		(0.001)
Imports, interm. prod., south		0.002		0.004**
		(0.003)		(0.001)
Exports, final prod., north		− 0.004		− 0.002
		(0.010)		(0.013)
Exports, interm. prod., north		− 0.001		− 0.010
		(0.005)		(0.008)
Exports, final prod., south		− 0.002		0.004
		(0.003)		(0.004)
Exports, interm. prod., south		0.004***		0.007**
		(0.001)		(0.003)
Constant	0.104***	0.104***	0.103***	0.101***
	(0.013)	(0.014)	(0.014)	(0.014)
Number of observations	20,878	19,391	20,866	19,380
Adjusted *R*^2^	0.045	0.045	0.045	0.048

Note: All regressions including time and individual fixed effects

Source: Panel data set constructed using data from FOS (2009a), EZV (2009), KOF (2005) and FOS (2009b)

*p < 0.10, **p < 0.05, ***p < 0.01

The estimates reported in Table 3 do not lend broad support for a positive relationship between the level of imports and the risk of becoming unemployed: Most coefficients of the import-level variables are not significantly different from zero. One exception at the 1% significance level is the coefficient of the 1-year lagged imports of final products from the South (fourth column): Individuals employed in an industry characterized by a 1% higher value of imports in this category encounter a 0.008% higher probability of becoming unemployed.

The results of the analogous estimations for first differences (i.e. rates of changes) in the import and export variables in a given industry are reported in Table 4. We neither find an unambiguous relationship between changes in imports and the risk of unemployment nor is any of the relationship significant on the 1% level. However, we find that the coefficients for a lagged increase in final as well as intermediate imports from the South are significantly different from zero (on the 5% level, fourth column). Note that the economic impact of this effect is small: A 1% increase in import value, denoted as 0.01 in the dataset, leads to an increase in the probability of becoming unemployed by 0.004%. On this background, the fact that the coefficients of intermediate export products to the South in columns (2) and (4)—0.004 and 0.007—are significantly different from zero (and positive) should not be overvalued.

### Sensitivity analyses

We finally try a number of different specifications to test the robustness of our results. Detailed results of these analyses are available from the Additional file 1 to this paper (Tables OA1 to OA5).

First, we replicate the results presented in Tables 2, 3 and 4 using the *logit regression model* (Additional file 1: Tables OA2 and OA3). Regarding the results in Table 2, the logit estimates confirm a relationship between import *levels* and the likelihood of low-skilled workers of becoming unemployed: Coefficients are significantly different from zero (at the 5% level) with a positive sign. Also, we can confirm sign and significance level for the individual socio-demographic variables included and reported in Table 2. Using a logit model with fixed effects, analogously to Tables 3 and 4, we do not find any significant effects of the trade variables, regardless of whether we use levels or first differences as explanatory variables.^18^ Hence, the logit estimations lead to qualitatively identical results as the linear regression model.

Second, we use the *employment status* (i.e. the information whether an individual is employed (0) or unemployed (1) in period *t*)—instead of the change of the employment status—as the dependent variable (summary statistics can be found in the second column of Table 1). As a start, we replicate the estimations described in Table 2 with the new dependent variable (see Additional file 1: Table OA4). Again, we can confirm positive coefficients regarding import *levels* interacted with low-skilled labour for lagged imports (significantly different from zero at the 5% level). Furthermore, we use trade levels and first differences as explanatory variables in a model with individual fixed effects and find results that are qualitatively similar to those in Tables 3 and 4. The results for the employment status as the dependent variable are reported in Additional file 1: Table OA5. Most coefficients are not significantly different from zero. One exception is, again, the lagged level of final imports from the South with a coefficient of 0.016 (significantly different from zero at the 1% level). However, we also find a negative coefficient for the lagged first differences of intermediate imports from the North (− 0.010, significantly different from zero at the 5% level), leaving us with an ambiguous result regarding the effect of imports on the status of employment.^19^

Third, and complementary to the analyses in Tables 3 and 4 (with again the change of the employment status as the dependent variable), we use *second differences* of the trade variables (e.g. [*IM*_*t*_ *−* (*IM*_*t −* 2_)/(*IM*_*t −* 2_)]) instead of first differences and *2-year lags* of trade levels instead of 1-year lags. All the results including the ones from Tables 3 and 4 are reported in Additional file 1: Table OA1. We find a negative coefficient for the second *differences* without lags of intermediate imports from the North (− 0.006, significantly different from zero on the 5% level) in column 14. Furthermore, a positive coefficient is found for intermediate import *levels* from the North lagged by 2 years (0.013, significantly different from zero on the 5% level) in column 6. All the other import coefficients are insignificantly different from zero.^20^ Thus, also in these regressions, we do not find unambiguous evidence for a positive relationship between imports and the probability of becoming unemployed.

## Conclusions

This paper has been sparked by the omnipresent public concern in many industrial countries that international trade through specialization and outsourcing may cause income losses and unemployment, particularly for low-skilled labour. The striking increase in the Swiss unemployment rate of low-skilled relative to high-skilled labour from 1991 to 2014—with virtually no changes of relative wages—motivated us to focus our research on the relationship between international trade and unemployment for Switzerland.

Our assessment of the Swiss case does not confirm the public concerns. The econometric analysis of a data set of roughly 30,000 workers in the Swiss manufacturing sector from 1991 to 2008, which we link with the Swiss foreign trade statistics, does not, overall, support the presumption that an *increase* in imports has a statistically significant (and positive) effect on the probability of individuals of becoming unemployed, irrespective of their skills. Thus, we seem to be left with other well-established factors such as the level of skills, temporary employment or the length of tenure to explain the individuals’ risk of unemployment. The startling rise in the relative unemployment rate of low-skilled labour and, at the same time, the somewhat comforting constant relative wage rate of low-skilled labour in Switzerland from 1991 to 2014 still remains to be explained. Obvious candidates to look at more carefully would, in our view, be a skill-biased technological change for the relative unemployment rate and the compositional change in immigration for the relative wage rate.^21^

Our investigation therefore only offers an initial basis for a more profound analysis of the labour market effects of trade or, more generally, of globalization for Switzerland. First, the fact that we find a weak (albeit small) positive relationship between low-skilled individuals working in industries characterized by a relatively high *level* of imports (particularly from the South) and the probability of their becoming unemployed may indicate something that we are not able to identify, given the limited statistical power of our data set which includes only a relatively small number of individuals who became unemployed. Second, we use exports as a control variable for (changes in) demand, because increasing imports have different effects on employment if they are combined with rising exports. This presents no problem as long as the domestic markets remain relatively small, which may, even in a small country such as Switzerland, not always be the case. If compatible data were available, a more sophisticated ratio could be used such as the import penetration ratio proposed by Autor et al. (2014) for the US industries.

Third, the fact that the individuals’ characteristics could only be linked to the two-digit SIC industry level, may even out a large amount of variation within industries: An individual’s employment status may be affected by imports on a sub-industry level, which might remain unobserved on the aggregated industry level. Also, and related to this, individuals employed in large multiproduct firms may be linked to an industry which is not really relevant to their actual occupation. Thus, an analysis based on more disaggregated, possibly even firm- or establishment-level, data may challenge our results.

On the other hand, this paper’s lack of findings in support of a strong positive relationship between import competition and the risk of unemployment could also be a consequence of the relatively low unemployment rate in Switzerland and the alleged high degree of flexibility in the Swiss labour market. If individuals lose their job because of import competition, but immediately find a new one, they never become unemployed. In this regard, it is interesting to note that our analysis of six announced mass-layoff cases in Swiss manufacturing due to globalization between 2001 and 2006 revealed exactly this situation: Only one quarter of the displaced workers were, in the end, dismissed by their companies and thus became, at least for a short term, unemployed (see Wyss, 2010). The others swiftly found a new job in the same or in another company or industry.

### Additional file

Additional file 1: **Table OA1.** Linear regressions of changes in employment status on trade variables using individual fixed effects. **Table OA2.** Logit regressions of changes in employment status on trade variables and individual characteristics, regression coefficients. **Table OA3.** Logit regressions of changes in employment status on trade variables using individual fixed effects, regression coefficients. **Table OA4.** Linear regressions of employment status on trade variables and individual characteristics, regression coefficients. **Table OA5.** Linear regressions of employment status on trade variables using individual fixed effects, regression coefficients. (DOCX 54 kb)

## Appendix

**Table 5:** Industry dummies for ICT intensity and GAV intensity

	Industry	ICT intensive	GAV intensive
1	Mining and quarrying	0	0
2	Manufacture of food products and beverages	0	0
3	Manufacture of textiles	0	0
4	Manufacture of wearing apparel	1	0
5	Manufacture of wood and of products of wood	0	1
6	Manufacture of paper and paper products	0	0
7	Publishing, printing and reproduction of recorded media	1	0
8	Manufacture of chemicals and chemical products	0	0
9	Manufacture of rubber and plastics products and other non-metallic mineral products	0	0
10	Manufacture of basic metals	0	1
11	Manufacture of fabricated metal products	0	0
12	Manufacture of machinery and equipment	1	1
13	Manufacture of office, accounting and computing machinery and other electrical machinery	1	0
14	Manufacture of radio, television and communication equipment and apparatus	1	1
15	Manufacture of medical, precision and optical instruments, watches and clocks	0	1
16	Manufacture of motor vehicles, trailers and semi-trailers	0	1
17	Furniture, rest of manufacturing	0	0

Source: Own composition based on KOF (2005) and FOS (2002). The ICT dummy equals 1 if the investment in information and communication technology is above the average of 16% of total investment. The GAV dummy equals 1 if the coverage by collective labour contracts is above the average of 36%

## Footnotes

1 Interestingly, this point of view is emphasized, for example, in an early document of the U.S. Department of State (1945) that formed the basis of the creation of the General Agreement on Tariffs and Trade (GATT). The title “Proposals of Expansion of World Trade and Employment” is revealing.

2 For early contributions see, for example, Berman et al. (1994), Borjas et al. (1991), Davis (1998a, 1998b), Feenstra (1998, 2010), Krugman (1995, 2000), Lawrence and Slaughter (1993), Leamer (1998, 2000) or Murphy and Welch (1991).

3 Whereas the overall effect on unemployment remains ambiguous or depends on parameters in these models, Dutt et al. (2009) predict a reduction in overall unemployment as a result of trade.

4 Moser et al. (2011) find a small (negative) effect from a reduction in the international competitiveness of firms on job flows for Germany, and more so on job creation rather than on job destruction.

5 See for example Feenstra and Hanson (1999), Hijzen et al. (2005) and OECD (2007) for a broad overview.

6 See Suarez (1998) and Müller, Marti and Nieuwkoop (2002) who focus on trade and wages. Other studies such as Sheldon (2007), Puhani (2003) and Arvanitis (2005) analyze shifts in supply and demand on the Swiss labour market, but do not explicitly investigate the effects of trade.

7 Note that, throughout this paper, high-skilled (H) is defined as people with tertiary education (ISCED 5-6: university, college of higher education (Fachhochschule) and school of higher education (Höhere Fachschule). Low-skilled (L) is defined as individuals with primary or lower secondary education (ISCED 1-2: mandatory education with no professional training qualification). Medium-skilled (M) is defined as individuals with upper secondary education (ISCED 3-4: professional education which, most importantly, includes completed apprenticeships).

8 Note that *U*_L_ is defined as the unemployment rate of the 25–64-year-olds with “below upper secondary education”, whereas *U*_H_ is defined as the unemployment rate of the 25–64-year-olds with “tertiary education”; see OECD (2007, 2015).

9 Interestingly, South Korea shows the lowest absolute rate of relative unemployment in 2014 despite the considerable increase reported in Fig. 2. On the other extreme, the Czech Republic shows a fall of the relative unemployment rate from 1991 to 2014, but remains the country with the highest ratio in 2014; note that, in 2014, *U*_L_ (*U*_H_) equaled 20.7% (2.6%) for this country (see OECD (2015, Table A5.4a)).

10 Baldwin (1994, p. 73) once called the view that trade affects the number of jobs as “utter nonsense from the medium- or long-run economic perspectives”. Davidson et al. (1999) would, however, add that in a trade model with labour market frictions this is, in principle, possible, and mainly an empirical question (p. 273).

11 Dutt et al., 2009, p. 33) emphasize a “fairly strong and robust empirical support (…) for the Ricardian prediction that trade openness and unemployment are negatively related across all countries”. The intuition is that trade raises productivity which increases the search effort of employees and employers that, in turn, reduces unemployment.

12 Note that, during this period, Switzerland or EFTA (to whom Switzerland belongs) established free trade agreements with approximately 20 countries (e.g. with Turkey (1992), Mexico (2001), South Korea (2006) and China (2014)), reached two bilateral agreements with the EU (1999, 2004) and was—through its free trade agreement (1972) and the two bilateral agreements with the EU—also affected by the enlargement of the EU by 13 new member countries in 2004, 2007 and 2013. Finally, there are approximately 30 countries (including China in 2001) that became additional members of the WTO, after its foundation in 1995 until 2008, and thus achieved improved mutual market access with Switzerland.

13 The deviation to all 33,000 individuals mentioned above is due to the fact that many individuals exhibit missing values in at least one of the variables of interest.

14 See Appendix: Table 5 regarding the assignment of individual industries. GAV stands for “Gesamt-Arbeits-Vertrag” and means collective bargaining contract; ICT stands for “Information and Communication Technology”.

15 Here and in the following we consider coefficients as significantly different from zero if they reach at least the 5% level.

16 Note that Feenstra and Hanson (2003) also base their analysis on annual changes broken down to final and intermediate imports. Anderton and Brenton (1999) differentiate between imports from industrial and low-wage countries. Based on the Swiss Trade Statistics, intermediates are defined as items in the category “raw materials”, “semi-finished products” and “intermediate goods”. An alternative definition based on input-output tables is currently not feasible as relevant statistics are not available. We also distinguish between imports from the North (industrial countries) and the South (developing countries).

17 Individuals remain in the same industry throughout the observed period. Hence, ICT and GAV variables are omitted when using individual fixed effects.

18 One may observe that the logit analysis implies a positive relationship (significantly different from zero at the 5% level) between low-skilled individuals working in GAV-industries (interacted variable) and their probability of becoming unemployed.

19 Also the relationship between the employment status and exports (level, change) remains ambiguous in the analysis.

20 Note that we also use 1-year leads of the trade variables as “placebo tests”. We refrain from showing those results in the Additional file 1 as we do not find any significant results.

21 For analyses of skill-biased technological change, see the seminal contributions by Berman et al. (1994, 1998) and Krugman (2000) as well as, for an attempt to disentangle trade and technology effects, Autor et al. (2015).
