# Supplementary material for: International trade and unemployment: towards an investigation of the Swiss case
Source: Swiss J Econ Stat. 2018 Jun 5;154(1):10. doi: 10.1186/s41937-017-0006-7 (PMC6214289; doi:10.1186/s41937-017-0006-7)
Supplement: Supplementary file 1 — Table OA1. Linear regressions of changes in employment status on trade variables using individual fixed effects. Table OA2. Logit regressions of changes in employment status on trade variables and individual characteristics, regression coefficients. Table OA3. Logit regressions of changes in employment status on trade variables using individual fixed effects, regression coefficients. Table OA4. Linear regressions of employment status on trade variables and individual characteristics, regression coefficients. Table OA5. Linear regressions of employment status on trade variables using individual fixed effects, regression coefficients. (DOCX 54 kb) [file 41937_2017_6_MOESM1_ESM.docx]

**Additional file 1**

**Table OA1: Linear Regressions of Changes in Employment Status on Trade Variables Using Individual Fixed Effects**

| **Dependent variable: Change in Employment Status** | | | | | | | | | | | | | | | | | | | | |
| --- | --- | --- | --- | --- | --- | --- | --- | --- | --- | --- | --- | --- | --- | --- | --- | --- | --- | --- | --- | --- |
|  | **Trade levels** | | **Trade levels lagged one period** | | **Trade levels lagged two periods** | |  | **Trade first differences** | | **Trade first differences lagged one period** | | **Trade first differences lagged two periods** | |  | **Trade second differences** | | **Trade second differences lagged one period** | | **Trade second differences lagged two periods** | |
|  | **(1)** | **(2)** | **(3)** | **(4)** | **(5)** | **(6)** |  | **(7)** | **(8)** | **(9)** | **(10)** | **(11)** | **(12)** |  | **(13)** | **(14)** | **(15)** | **(16)** | **(17)** | **(18)** |
| IM total | 0.016 |  | -0.002 |  | 0.043 |  |  | 0.013 |  | -0.009 |  | -0.011 |  |  | 0.002 |  | -0.007 |  | 0.014 |  |
|  | (0.022) |  | (0.019) |  | (0.026) |  |  | (0.018) |  | (0.015) |  | (0.019) |  |  | (0.010) |  | (0.014) |  | (0.020) |  |
| EX total | -0.036* |  | 0.004 |  | -0.015 |  |  | -0.025 |  | 0.010 |  | 0.036** |  |  | -0.010 |  | 0.008 |  | 0.002 |  |
|  | (0.018) |  | (0.013) |  | (0.016) |  |  | (0.015) |  | (0.007) |  | (0.013) |  |  | (0.007) |  | (0.011) |  | (0.016) |  |
| IM final prod., north |  | 0.013 |  | 0.003 |  | 0.023 |  |  | 0.008 |  | 0.002 |  | -0.002 |  |  | 0.006 |  | 0.001 |  | 0.016* |
|  |  | (0.014) |  | (0.015) |  | (0.016) |  |  | (0.014) |  | (0.015) |  | (0.014) |  |  | (0.004) |  | (0.009) |  | (0.008) |
| IM interm. prod., north |  | -0.003 |  | 0.006* |  | 0.013** |  |  | -0.004 |  | -0.005* |  | -0.004 |  |  | -0.006** |  | 0.001 |  | 0.005 |
|  |  | (0.005) |  | (0.003) |  | (0.006) |  |  | (0.004) |  | (0.003) |  | (0.004) |  |  | (0.002) |  | (0.002) |  | (0.004) |
| IM final prod., south |  | 0.002 |  | 0.008*** |  | 0.009* |  |  | -0.000 |  | 0.004** |  | 0.005* |  |  | 0.001 |  | 0.003 |  | -0.002 |
|  |  | (0.003) |  | (0.003) |  | (0.005) |  |  | (0.001) |  | (0.001) |  | (0.003) |  |  | (0.001) |  | (0.002) |  | (0.005) |
| IM interm. prod., south |  | 0.002 |  | -0.000 |  | -0.002 |  |  | 0.002 |  | 0.004** |  | 0.002 |  |  | 0.005 |  | -0.001 |  | -0.000 |
|  |  | (0.006) |  | (0.003) |  | (0.005) |  |  | (0.003) |  | (0.001) |  | (0.003) |  |  | (0.004) |  | (0.002) |  | (0.005) |
| EX final prod., north |  | -0.002 |  | 0.001 |  | -0.020 |  |  | -0.004 |  | -0.002 |  | 0.016 |  |  | -0.002 |  | 0.003 |  | -0.004 |
|  |  | (0.012) |  | (0.013) |  | (0.018) |  |  | (0.010) |  | (0.013) |  | (0.011) |  |  | (0.007) |  | (0.011) |  | (0.014) |
| EX interm. prod., north |  | -0.019* |  | -0.012 |  | -0.013 |  |  | -0.001 |  | -0.010 |  | 0.001 |  |  | -0.006 |  | -0.001 |  | 0.002 |
|  |  | (0.009) |  | (0.009) |  | (0.014) |  |  | (0.005) |  | (0.008) |  | (0.006) |  |  | (0.005) |  | (0.003) |  | (0.004) |
| EX final prod., south |  | -0.009 |  | 0.001 |  | 0.005 |  |  | -0.002 |  | 0.004 |  | 0.005 |  |  | -0.001 |  | -0.001 |  | 0.004 |
|  |  | (0.008) |  | (0.006) |  | (0.014) |  |  | (0.003) |  | (0.004) |  | (0.004) |  |  | (0.004) |  | (0.003) |  | (0.005) |
| EX interm. prod., south |  | 0.010** |  | -0.001 |  | -0.004 |  |  | 0.004*** |  | 0.007** |  | -0.001 |  |  | 0.005** |  | -0.000 |  | -0.004 |
|  |  | (0.004) |  | (0.004) |  | (0.006) |  |  | (0.001) |  | (0.003) |  | (0.004) |  |  | (0.002) |  | (0.002) |  | (0.004) |
| Constant | 0.15*** | 0.12*** | 0.10*** | 0.10*** | 0.04*** | 0.09*** |  | 0.10*** | 0.10*** | 0.10*** | 0.10*** | 0.09*** | 0.10*** |  | 0.11*** | 0.11*** | 0.09*** | 0.09*** | 0.07*** | 0.07*** |
|  | (0.044) | (0.028) | (0.043) | (0.045) | (0.057) | (0.040) |  | (0.013) | (0.014) | (0.014) | (0.014) | (0.012) | (0.013) |  | (0.016) | (0.017) | (0.011) | (0.011) | (0.009) | (0.010) |
| No. obs. | 20,928 | 19,438 | 20,895 | 19,406 | 10,822 | 10,025 |  | 20,878 | 19,391 | 20,866 | 19,380 | 10,815 | 10,020 |  | 20,928 | 19,438 | 19,877 | 18,405 | 10,164 | 9,381 |
| Adjusted R2 | 0.045 | 0.047 | 0.045 | 0.047 | 0.045 | 0.050 |  | 0.045 | 0.045 | 0.045 | 0.048 | 0.046 | 0.050 |  | 0.044 | 0.047 | 0.047 | 0.050 | 0.049 | 0.054 |
| Note: All regressions including time and individual fixed effects. | | | | | | | | | | | | | | | | | | | | |

Source: Panel data set constructed using data from FOS (2009a), EZV (2009), KOF (2005) and FOS (2009b). Note that the results shown in columns (1) to (4) and (7) to (10) are the ones found in Table 3 and 4 in the paper, respectively. We repeat them here for convenience

**p*<0.10, ***p*<0.05, ****p*<0.01

**Table OA2: Logit Regressions of Changes in Employment Status on Trade Variables and Individual Characteristics, Regression Coefficients**

| **Dependent variable: Change in Employment Status** | | | | | | | |
| --- | --- | --- | --- | --- | --- | --- | --- |
|  | **No trade covariates** |  | **Trade levels** | **Trade levels, lagged** |  | **Trade first diff.** | **Trade first diff., lagged** |
|  | **(1)** |  | **(2)** | **(3)** |  | **(4)** | **(5)** |
| **Trade Covariates:** |  |  |  |  |  |  |  |
| Imports |  |  | 0.721 | 0.601 |  | -0.019 | 1.514 |
|  |  |  | (0.967) | (0.952) |  | (1.729) | (1.179) |
| Exports |  |  | 0.011 | -0.059 |  | 1.077 | -1.045 |
|  |  |  | (0.863) | (0.870) |  | (1.901) | (1.704) |
| Imports*low-skilled |  |  | 0.667** | 0.628** |  | -0.011 | -0.346 |
|  |  |  | (0.271) | (0.266) |  | (1.646) | (1.110) |
| Imports*medium-skilled |  |  | 0.143 | 0.094 |  | 0.496 | -1.488 |
|  |  |  | (0.267) | (0.273) |  | (1.571) | (1.239) |
| Exports*low-skilled |  |  | -0.401* | -0.375* |  | -0.617 | 0.320 |
|  |  |  | (0.223) | (0.219) |  | (2.145) | (1.468) |
| Exports*medium-skilled |  |  | -0.116 | -0.090 |  | -0.691 | 0.898 |
|  |  |  | (0.198) | (0.199) |  | (1.536) | (1.501) |
| **Industry characteristics:** |  |  |  |  |  |  |  |
| ICT intensive | -0.396 |  | 0.089 | 0.224 |  | -0.339 | -0.379 |
|  | (0.559) |  | (1.376) | (1.414) |  | (0.578) | (0.573) |
| ICT intensive*low-skilled | 0.050 |  | 0.201 | 0.204 |  | 0.057 | 0.074 |
|  | (0.297) |  | (0.308) | (0.317) |  | (0.302) | (0.310) |
| ICT intensive*medium-skilled | -0.117 |  | -0.081 | -0.091 |  | -0.125 | -0.086 |
|  | (0.279) |  | (0.316) | (0.319) |  | (0.263) | (0.270) |
| GAV | -0.259 |  | -0.393 | -0.542 |  | -0.326 | -0.327 |
|  | (0.569) |  | (1.309) | (1.367) |  | (0.586) | (0.579) |
| GAV*low-skilled | 0.611** |  | 0.563** | 0.575** |  | 0.607** | 0.626** |
|  | (0.273) |  | (0.277) | (0.279) |  | (0.271) | (0.283) |
| GAV*medium-skilled | 0.385 |  | 0.389 | 0.386 |  | 0.350 | 0.378 |
|  | (0.344) |  | (0.371) | (0.371) |  | (0.359) | (0.344) |
| **Worker and job characteristics:** |  |  |  |  |  |  |  |
| Low-skilled | 0.557*** |  | -0.044 | 0.005 |  | 0.604** | 0.547** |
|  | (0.208) |  | (0.278) | (0.289) |  | (0.257) | (0.275) |
| Medium-skilled | 0.339*** |  | 0.253 | 0.315 |  | 0.370** | 0.354* |
|  | (0.129) |  | (0.395) | (0.391) |  | (0.182) | (0.182) |
| Foreigner | 0.540*** |  | 0.531*** | 0.525*** |  | 0.516*** | 0.516*** |
|  | (0.115) |  | (0.111) | (0.110) |  | (0.115) | (0.117) |
| Age | -0.130*** |  | -0.130*** | -0.132*** |  | -0.135*** | -0.134*** |
|  | (0.031) |  | (0.032) | (0.032) |  | (0.030) | (0.031) |
| Age^2 | 0.002*** |  | 0.002*** | 0.002*** |  | 0.002*** | 0.002*** |
|  | (0.000) |  | (0.000) | (0.000) |  | (0.000) | (0.000) |
| Female | 0.131 |  | 0.130 | 0.123 |  | 0.136 | 0.138 |
|  | (0.143) |  | (0.143) | (0.143) |  | (0.144) | (0.148) |
| Married | -0.454*** |  | -0.449*** | -0.442*** |  | -0.413*** | -0.421*** |
|  | (0.102) |  | (0.104) | (0.103) |  | (0.100) | (0.098) |
| Widowed | -1.133** |  | -1.128** | -1.126** |  | -1.101** | -1.098** |
|  | (0.551) |  | (0.544) | (0.543) |  | (0.546) | (0.541) |
| Separated | 0.287** |  | 0.290** | 0.305** |  | 0.330*** | 0.326*** |
|  | (0.132) |  | (0.133) | (0.130) |  | (0.125) | (0.124) |
| Parttime worker | 0.419*** |  | 0.422*** | 0.417*** |  | 0.412*** | 0.411*** |
|  | (0.111) |  | (0.110) | (0.111) |  | (0.113) | (0.111) |
| Temporary worker | 1.629*** |  | 1.651*** | 1.640*** |  | 1.619*** | 1.616*** |
|  | (0.141) |  | (0.142) | (0.143) |  | (0.138) | (0.142) |
| Short tenure (<1 year) | 3.338*** |  | 3.327*** | 3.363*** |  | 3.376*** | 3.368*** |
|  | (0.143) |  | (0.142) | (0.143) |  | (0.145) | (0.144) |
| Medium tenure (1 year to <5 years) | 1.040*** |  | 1.036*** | 1.045*** |  | 1.050*** | 1.048*** |
|  | (0.066) |  | (0.066) | (0.065) |  | (0.068) | (0.068) |
| Constant | -3.207*** |  | -5.623** | -5.031* |  | -3.215*** | -3.254*** |
|  | (1.168) |  | (2.607) | (2.679) |  | (1.195) | (1.183) |
| Number of observations | 20,928 |  | 20,928 | 20,895 |  | 20,878 | 20,866 |
| Pseudo R2 | 0.190 |  | 0.193 | 0.194 |  | 0.193 | 0.193 |
| Note: All regressions including time and industry fixed effects. | | | | | | | |

Source: Panel data set constructed using data from FOS (2009a), EZV (2009), KOF (2005) and FOS (2009b)

**p*<0.10, ***p*<0.05, ****p*<0.01**Table OA3: Logit Regressions of Changes in Employment Status on Trade Variables Using Individual Fixed Effects, Regression Coefficients**

| **Dependent variable: Change in Employment Status** | | | | | | | | |
| --- | --- | --- | --- | --- | --- | --- | --- | --- |
|  | **Trade levels** | | **Trade levels, lagged** | | **Trade first differences** | | **Trade first differences, lagged** | |
|  | **(1)** | **(2)** | **(3)** | **(4)** | **(5)** | **(6)** | **(7)** | **(8)** |
| Imports, total | -1.608 |  | 1.698 |  | -1.125 |  | -0.931 |  |
|  | (4.921) |  | (4.787) |  | (3.127) |  | (3.494) |  |
| Exports, total | -0.871 |  | -3.723 |  | 1.246 |  | 0.234 |  |
|  | (4.499) |  | (4.904) |  | (3.752) |  | (4.299) |  |
| Imports, final prod., north |  | 1.789 |  | 7.110 |  | -0.971 |  | 2.580 |
|  |  | (4.485) |  | (5.669) |  | (3.352) |  | (4.769) |
| Imports, interm. prod., north |  | -0.227 |  | -2.817 |  | 0.497 |  | -0.450 |
|  |  | (1.715) |  | (3.521) |  | (1.355) |  | (1.689) |
| Imports, final prod., south |  | 0.924 |  | 1.118 |  | 0.274 |  | 0.288 |
|  |  | (0.998) |  | (1.340) |  | (0.542) |  | (0.677) |
| Imports, interm. prod., south |  | 0.904 |  | -1.798 |  | 0.391 |  | -0.363 |
|  |  | (1.599) |  | (2.445) |  | (0.817) |  | (0.963) |
| Exports, final prod., north |  | -3.218 |  | -2.992 |  | -1.370 |  | -0.437 |
|  |  | (3.941) |  | (4.188) |  | (2.370) |  | (2.959) |
| Exports, interm. prod., north |  | 0.290 |  | 5.614 |  | -1.122 |  | 0.436 |
|  |  | (4.001) |  | (4.880) |  | (2.893) |  | (3.432) |
| Exports, final prod., south |  | 2.067 |  | -2.766 |  | 2.270 |  | -0.275 |
|  |  | (2.666) |  | (3.482) |  | (1.952) |  | (1.658) |
| Exports, interm. prod., south |  | 1.118 |  | -0.817 |  | 0.707 |  | 0.146 |
|  |  | (1.585) |  | (1.929) |  | (0.883) |  | (1.003) |
| Number of observations | 459 | 427 | 456 | 424 | 452 | 420 | 452 | 420 |
| Pseudo R2 | 0.758 | 0.753 | 0.757 | 0.760 | 0.754 | 0.750 | 0.754 | 0.745 |
| Note: All regressions including time and individual fixed effects. | | | | | | | | |

Source: Panel data set constructed using data from FOS (2009a), EZV (2009), KOF (2005) and FOS (2009b)

**p*<0.10, ***p*<0.05, ****p*<0.01

**Table OA4: Linear Regressions of Employment Status on Trade Variables and Individual Characteristics, Regression Coefficients**

| **Dependent variable: Employment Status** | | | | | | | |
| --- | --- | --- | --- | --- | --- | --- | --- |
|  | **No trade covariates** |  | **Trade levels** | **Trade levels, lagged** |  | **Trade first diff.** | **Trade first diff., lagged** |
|  | **(1)** |  | **(2)** | **(3)** |  | **(4)** | **(5)** |
| **Trade Covariates:** |  |  |  |  |  |  |  |
| Imports |  |  | -0.007 | 0.015 |  | -0.029 | 0.022 |
|  |  |  | (0.020) | (0.023) |  | (0.042) | (0.030) |
| Exports |  |  | 0.004 | 0.003 |  | 0.019 | -0.006 |
|  |  |  | (0.021) | (0.023) |  | (0.032) | (0.031) |
| Imports*low-skilled |  |  | 0.013* | 0.020** |  | 0.029 | -0.013 |
|  |  |  | (0.007) | (0.008) |  | (0.062) | (0.051) |
| Imports*medium-skilled |  |  | 0.005 | 0.003 |  | -0.005 | -0.023 |
|  |  |  | (0.004) | (0.005) |  | (0.027) | (0.029) |
| Exports*low-skilled |  |  | -0.008 | -0.012** |  | 0.026 | 0.004 |
|  |  |  | (0.005) | (0.006) |  | (0.046) | (0.037) |
| Exports*medium-skilled |  |  | -0.002 | -0.002 |  | 0.002 | 0.005 |
|  |  |  | (0.003) | (0.004) |  | (0.022) | (0.031) |
| **Industry characteristics:** |  |  |  |  |  |  |  |
| ICT intensive | 0.016 |  | -0.011 | 0.055 |  | 0.013 | -0.002 |
|  | (0.024) |  | (0.044) | (0.076) |  | (0.025) | (0.028) |
| ICT intensive*low-skilled | -0.004 |  | -0.002 | 0.003 |  | -0.003 | 0.002 |
|  | (0.008) |  | (0.008) | (0.009) |  | (0.008) | (0.009) |
| ICT intensive*medium-skilled | -0.002 |  | -0.001 | 0.003 |  | -0.002 | 0.002 |
|  | (0.004) |  | (0.004) | (0.006) |  | (0.004) | (0.006) |
| GAV | -0.033 |  | 0.008 | 0.018 |  | -0.032 | -0.024 |
|  | (0.023) |  | (0.046) | (0.049) |  | (0.023) | (0.020) |
| GAV*low-skilled | 0.015** |  | 0.016** | 0.019** |  | 0.014** | 0.015** |
|  | (0.007) |  | (0.007) | (0.008) |  | (0.006) | (0.007) |
| GAV*medium-skilled | 0.004 |  | 0.003 | 0.001 |  | 0.004 | 0.002 |
|  | (0.004) |  | (0.005) | (0.006) |  | (0.005) | (0.006) |
| **Worker and job characteristics:** |  |  |  |  |  |  |  |
| Low-skilled | 0.013*** |  | 0.001 | -0.007 |  | 0.009 | 0.010 |
|  | (0.005) |  | (0.008) | (0.009) |  | (0.006) | (0.007) |
| Medium-skilled | 0.007*** |  | 0.001 | 0.004 |  | 0.008** | 0.008** |
|  | (0.003) |  | (0.005) | (0.007) |  | (0.003) | (0.003) |
| Foreigner | 0.011*** |  | 0.011*** | 0.011** |  | 0.011*** | 0.012*** |
|  | (0.004) |  | (0.004) | (0.005) |  | (0.004) | (0.005) |
| Age | -0.002* |  | -0.002* | -0.003** |  | -0.002* | -0.003** |
|  | (0.001) |  | (0.001) | (0.001) |  | (0.001) | (0.001) |
| Age^2 | 0.000** |  | 0.000** | 0.000** |  | 0.000** | 0.000** |
|  | (0.000) |  | (0.000) | (0.000) |  | (0.000) | (0.000) |
| Female | 0.000 |  | 0.001 | 0.000 |  | 0.000 | 0.000 |
|  | (0.002) |  | (0.002) | (0.004) |  | (0.002) | (0.004) |
| Married | -0.004 |  | -0.004 | -0.010*** |  | -0.004 | -0.010*** |
|  | (0.003) |  | (0.003) | (0.004) |  | (0.003) | (0.004) |
| Widowed | -0.019*** |  | -0.019*** | -0.025*** |  | -0.019*** | -0.025*** |
|  | (0.006) |  | (0.006) | (0.009) |  | (0.006) | (0.009) |
| Separated | 0.012** |  | 0.012** | 0.007 |  | 0.012** | 0.007 |
|  | (0.006) |  | (0.005) | (0.005) |  | (0.006) | (0.005) |
| Parttime worker | 0.011** |  | 0.010** | 0.011** |  | 0.010** | 0.011** |
|  | (0.005) |  | (0.004) | (0.005) |  | (0.004) | (0.005) |
| Temporary worker | 0.064*** |  | 0.064*** | 0.111*** |  | 0.064*** | 0.111*** |
|  | (0.019) |  | (0.019) | (0.029) |  | (0.019) | (0.029) |
| Short tenure (<1 year) | 0.112*** |  | 0.112*** | 0.186*** |  | 0.112*** | 0.186*** |
|  | (0.031) |  | (0.031) | (0.046) |  | (0.031) | (0.046) |
| Medium tenure (1 year to <5 years) | 0.024*** |  | 0.024*** | 0.024*** |  | 0.024*** | 0.024*** |
|  | (0.007) |  | (0.007) | (0.008) |  | (0.007) | (0.008) |
| Constant | 0.078 |  | 0.072 | 0.340 |  | 0.082 | 0.411 |
|  | (0.079) |  | (0.091) | (0.267) |  | (0.081) | (0.267) |
| Number of observations | 40,875 |  | 40,875 | 21,480 |  | 40,875 | 21,480 |
| Adjusted R2 | 0.061 |  | 0.061 | 0.088 |  | 0.061 | 0.087 |
| Note: All regressions including time and industry fixed effects. | | | | | | | |

Source: Panel data set constructed using data from FOS (2009a), EZV (2009), KOF (2005) and FOS (2009b)

**p*<0.10, ***p*<0.05, ****p*<0.01**Table OA5: Linear Regressions of Employment Status on Trade Variables Using Individual Fixed Effects, Regression Coefficients**

| **Dependent variable: Employment Status** | | | | | | | | |
| --- | --- | --- | --- | --- | --- | --- | --- | --- |
|  | **Trade levels** | | **Trade levels, lagged** | | **Trade first differences** | | **Trade first differences, lagged** | |
|  | **(1)** | **(2)** | **(3)** | **(4)** | **(5)** | **(6)** | **(7)** | **(8)** |
| IM, total | -0.051 |  | 0.019 |  | -0.040* |  | 0.000 |  |
|  | (0.030) |  | (0.021) |  | (0.020) |  | (0.014) |  |
| EX, total | -0.002 |  | -0.020 |  | 0.030* |  | -0.005 |  |
|  | (0.031) |  | (0.018) |  | (0.014) |  | (0.009) |  |
| IM, final prod., north |  | -0.035* |  | 0.011 |  | -0.017 |  | 0.014 |
|  |  | (0.018) |  | (0.013) |  | (0.018) |  | (0.013) |
| IM, interm. prod., north |  | -0.003 |  | 0.003 |  | -0.003 |  | -0.010** |
|  |  | (0.009) |  | (0.004) |  | (0.004) |  | (0.005) |
| IM, final prod., south |  | 0.005 |  | 0.016*** |  | -0.000 |  | 0.004* |
|  |  | (0.004) |  | (0.004) |  | (0.002) |  | (0.002) |
| IM, interm. prod., south |  | 0.000 |  | 0.002 |  | -0.001 |  | 0.002 |
|  |  | (0.006) |  | (0.006) |  | (0.003) |  | (0.002) |
| EX, final prod., north |  | 0.006 |  | -0.015 |  | 0.021** |  | -0.010 |
|  |  | (0.016) |  | (0.015) |  | (0.008) |  | (0.015) |
| EX, interm. prod., north |  | -0.045*** |  | -0.018 |  | -0.018*** |  | -0.013* |
|  |  | (0.008) |  | (0.012) |  | (0.006) |  | (0.007) |
| EX, final prod., south |  | 0.006 |  | 0.001 |  | 0.006 |  | 0.004 |
|  |  | (0.008) |  | (0.007) |  | (0.006) |  | (0.007) |
| EX, interm. prod., south |  | 0.023** |  | 0.007 |  | 0.005* |  | 0.012*** |
|  |  | (0.007) |  | (0.005) |  | (0.003) |  | (0.003) |
| Constant | 0.160** | 0.146** | 0.109** | 0.147** | 0.037 | 0.039 | 0.111*** | 0.108*** |
|  | (0.067) | (0.050) | (0.046) | (0.056) | (0.024) | (0.025) | (0.017) | (0.018) |
| No. obs. | 40,875 | 38,104 | 21,480 | 19,959 | 40,875 | 38,104 | 21,480 | 19,959 |
| Adjusted R2 | 0.006 | 0.007 | 0.033 | 0.036 | 0.006 | 0.007 | 0.033 | 0.036 |
| Note: All regressions including time and individual fixed effects. | | | | | | | | |

Source: Panel data set constructed using data from FOS (2009a), EZV (2009), KOF (2005) and FOS (2009b)

**p*<0.10, ***p*<0.05, ****p*<0.01
